# Neurofilament Light Chain serum levels after Hypoxia-Ischemia in a newborn piglet model

**DOI:** 10.3389/fped.2022.1068380

**Published:** 2023-01-09

**Authors:** Kasper Jacobsen Kyng, Sven Wellmann, Verena Lehnerer, Lærke Hjøllund Hansen, Jens Kuhle, Tine Brink Henriksen

**Affiliations:** ^1^ Aarhus University Hospital and Aarhus University, Department of Pediatrics and Adolescent Medicine and Department of Clinical Medicine, Aarhus, Denmark; ^2^ Department of Neonatology, University Children’s Hospital Regensburg (KUNO), Hospital St. Hedwig of the Order of St. John, University of Regensburg, Regensburg, Germany; ^3^ Neurologic Clinic and Policlinic, MS Center and Research Center for Clinical Neuroimmunology and Neuroscience Basel (RC2NB), Departments of Biomedicine and Clinical Research, University Hospital Basel, Basel, Switzerland

**Keywords:** NfL, hypoxia-ischemia, hypoxic-ischemic encephalopathy, newborn brain injury, biomarker, animal model

## Abstract

**Aim:**

Neurofilament light Chain (NfL) is a promising brain injury biomarker which may assist diagnosis and prognostication in hypoxic-ischemic encephalopathy (HIE). The aim of this study was to investigate serum NfL levels after hypoxia-ischemia (HI) in a newborn piglet model. Second, to characterize the influence of sex, weight, and treatment with remote ischemic postconditioning (RIPC) on NfL and the correlation between NfL, brain imaging and histologic brain injury.

**Methods:**

We used serum from 48 newborn piglets of both sexes subjected to 45 min of global HI, and 4 sham piglets. Blood was collected pre-HI, 2 h post-HI and 72 h post-HI. NfL was measured by single-molecule array (Simoa™). We analysed the temporal profile of NfL after HI, and correlations between NfL, magnetic resonance spectroscopy brain Lac/NAA ratios and histologic brain injury 72 h after HI.

**Results:**

Median (IQR) NfL levels were: pre-HI: 66 pg/ml (45–87), 2 h post-HI: 105 pg/ml (77–140), and 72 h post-HI: 380 pg/ml (202–552). The increase in NfL after HI was statistically significant (*p* < 0.0001, mixed-effects ANOVA). Median NfL levels in sham animals were 41.4 pg/ml at baseline and 92.4 pg/ml at 72 h (*p* = 0.11, paired t-test). Neither sex, nor treatment with RIPC influenced NfL levels. Weight had a small, not biologically important, influence. NfL levels at 72 h were moderately correlated with histologic brain injury and brain Lac/NAA ratios. NfL 72 h post-HI > 330 pg/ml had a sensitivity of 89% (95% CI, 57%–99%) and a specificity of 52% (95% CI, 34%–69%) for predicting basal ganglia Lac/NAA ratio in the highest quartile. NfL 72 h post-HI > 445 pg/ml had a sensitivity of 90% (95% CI, 60%–99%) and a specificity of 74% (95% CI, 58%–86%) for predicting cortical brain histopathology injury in the highest quartile.

**Conclusion:**

NfL increased after HI, with the largest values at 72 h post-HI. Early NfL was sensitive but not very specific, whereas NfL at 72 h was both highly sensitive and specific for exposure to moderate-severe HI in this model of HI-induced brain injury. This was supported by a moderate correlation of NfL at 72 h with brain Lac/NAA ratio and histopathology.

## Introduction

Perinatal hypoxia-ischemia (HI) can result in hypoxic-ischemic encephalopathy (HIE), which affects 1–8 per 1,000 live births and is associated with significant neurologic morbidity and mortality (1). Currently, no biochemical brain injury biomarkers are used in standard of care for HIE. Diagnosis of neuronal injury is based on non-specific clinical signs of encephalopathy, and there is a need for biomarkers of brain injury to improve diagnosis, guide treatment decisions, monitor treatment response and assist the development of new neuroprotective treatments (2, 3). Neurofilament Light chain (NfL) may be a useful biomarker for newborn brain injury. NfL is a subunit of the neurofilament scaffolding proteins present in myelinated axons and the CNS. After axonal injury, NfL is released to the interstitial fluid and can be measured in both CSF and blood. NfL levels increase with acute or chronic neuronal injury, including stroke and traumatic brain injury in adults (4). NfL can be measured in newborns and has been found to be higher after vaginal delivery than after caesarean section and further increased in newborns suffering from intraventricular haemorrhage (5, 6). Clinical interpretation of NfL in HIE is challenged by competing aetiologies of neonatal encephalopathy and uncertain timing of hypoxic-ischemic events. The piglet model is uniquely suited to study biomarkers of brain injury, as serum protein levels can be compared before and after HI in the same animal, ruling out other inter-individual contributing factors (7). Thus, the aim of this study was to characterize baseline and post-HI NfL serum levels in a clinically relevant piglet model of moderate to severe hypoxia-ischemia and subsequent brain injury. Further, to identify NfL cut-off values for identifying piglets exposed to the moderate-severe HI and associated high risk of brain injury in the model. Second, we aimed to investigate if sex and weight influence NfL values, and whether treatment with remote ischemic postconditioning (RIPC) affects NfL levels. Finally, we examined how NfL correlates with brain imaging and histologic brain injury and whether NfL can predict brain Lactate/N-acetylaspartate ratio k(Lac/NAA) measured by magnetic resonance spectroscopy (MRS) and histologic brain injury.

## Materials and methods

### Animal model

The study was approved by the Danish Animal Experiments Inspectorate (2012–15–2934–00036) and conducted in accordance with national ethics and animal welfare guidelines. Reporting complies with ARRIVE 2.0 guidelines for reporting of animal research. The porcine brain is seen as an appropriate gyrencephalic model for the brain of human infants (8). As in humans, the pig brain develops perinatally with a peak growth rate around the time of birth (9). We used serum from 48 Danish Landrace piglets of both sexes subjected to global HI and 4 sham piglets exposed to anaesthesia and normoxic mechanical ventilation only in a previously published study designed to evaluate the effects of RIPC (10). Piglets were born at term, weighed 1674 g (range 800 g–2500 g), were 21 h old (range 18 h–25 h) at experiment start and 46% were male. Global HI was induced as previously described (11–13). Studies were started at 8 am. Briefly, anaesthesia was initiated with 1%–2% sevoflurane delivered *via* a mask (Air Liquide, France). For intubation, 5 mg/kg propofol, 10 µg/kg fentanyl and 1 mg/kg rocuronium was given i.v. Anaesthesia was maintained by i.v. propofol 4–12 mg/kg/h and fentanyl 10 µg/kg/h. Sevoflurane was discontinued after intubation and maintenance anaesthesia given by continuous i.v. infusion of propofol (4–12 mg/kg/h) and fentanyl (10 µg/kg/h), then weaned in the hours after HI. Mechanical ventilation was adjusted to maintain P_a_CO_2_ at 4.5–5.5 kPa. Umbilical venous 5 Fr and arterial 3.5 Fr catheters were placed. The umbilical venous catheter was used for fluid and drug administration. An i.v. infusion of 5% dextrose with 0.45% NaCl was started at 10 ml/kg/h. During and after HI the infusion rate was reduced to 5 ml/kg/h and adjusted to maintain blood glucose between 3 and 10 mmol/L. Procaine benzylpenicillin (15.000 IU/kg s.c.) was given once daily. A probe measuring oxygen saturation was placed on a leg. The piglet was placed supine on a heated inflatable air mattress to maintain a normal rectal temperature at 38.5 ± 0.5°C. ECG electrodes were placed for continuous heart rate monitoring. Mean arterial blood pressure (MABP) was measured continuously using the arterial umbilical catheter. Arterial blood gases (P_a_O_2_, P_a_CO_2_, pH, standard base excess) and serum electrolytes, blood glucose and lactate were analysed at baseline, after 30 min of HI, at the end of 45 min HI, 3 h after HI-start and 72 h after HI-start (Radiometer Medical, Denmark). A single channel biparietal aEEG was continuously recorded during HI, the recovery period and again at day 3 (Niculet monitor, Natus Medical Incorporated, USA).

### Hypoxia-ischemia

After 60 min stabilization, global hypoxia was induced by switching to a 4% O_2_ in nitrogen (N_2_) gas mixture for 45 min and lowering the respiratory rate to 16 to further decrease both the mean airway pressure and the oxygenation, while also decreasing CO_2_ removal to mimic the universal decrease in gas exchange in a perinatal HI event. Once the aEEG trace upper margin was below 7 µV, the inspired oxygen fraction (F_i_O_2_) was adjusted to maintain the aEEG trace between 3 and 5 µV. We aimed for 10 min with mean arterial blood pressure below 70% of the baseline, and if needed F_i_O_2_ was lowered further. After 45 min F_i_O_2_ was increased to 21%. Seizures (clinical or electroencephalographic lasting >10 min) were treated with phenobarbitone 20 mg/kg, repeat phenobarbitone 20 mg/kg, and midazolam 0.5 mg/kg. One hour post HI piglets were randomised to four times five minutes of RIPC by external occlusion of blood supply to both hind limbs or supportive treatment only. RIPC was induced one hour after HI by occluding blood flow to both hind limbs using an external plastic strip for 5 min followed by 5 min reperfusion, repeated for a total of 4 cycles of occlusion/reperfusion. The strip was tightened until complete occlusion of arterial blood flow was verified by Doppler ultrasound indicating supra-systolic pressure. The exact pressure applied by the plastic strip was not measured. Animal technicians caring for the animals were blinded to randomization group at all times. Investigators were blinded to randomization group when assessing outcomes.

### Seventy-two-hour survival period

After HI, anaesthesia was weaned, and piglets extubated. During the survival period piglets were kept in pairs and cared for at a dedicated animal facility. The piglets received daily antibiotics and were fed every two hours. After 72 h, the piglets were anaesthetized for MRI/MRS scanning, then euthanized by a lethal dose of pentobarbital.

### Nfl analysis

Blood was collected from the umbilical arterial catheter, under anaesthesia, at baseline/pre-HI (NfL-base), 2 h post-HI (NfL-2 h) and 72 h post-HI (NfL-72 h), and frozen at −80°C until analysis. Sham piglets were only sampled at baseline and 72 h, as without HI, no significant change in blood tests was expected to occur between baseline and 2 h. Thus 2 h blood samples were not available for sham piglets. Serum concentrations of NfL were determined with a Simoa assay, using capture monoclonal antibody (mAB) 47:3 and biotinylated detector mAB 2:1 (UmanDiagnostics, Umea, Sweden), as previously described (5). Calibrators (neat) and serum samples (1:4 dilution) were measured in duplicates. Batch-prepared calibrators were stored at −80°C. Interassay coefficients of variation were <7%.

### Brain Lac/NAA and neuropathology

MRI/MRS scans and neuropathology was acquired at 72 h after HI as previously described (10). A scout T2-weighted spin-echo sequence was initially employed to locate the brain and was used to prepare subsequent sequences. Next, a diffusion-weighted spin-echo sequence with fat-saturation encoding was employed (parameters: FOV = 160 × 160 mm^2^, resolution = 1.1 × 1.1 × 3.0 mm^3^, TR = 3300 ms, TE = 108 ms, and diffusion b-values = 0 and 800 s/mm^2^). A decrease in the apparent diffusion coefficient (ADC) reflects cellular swelling. A STEAM-based fat-saturated spectroscopy protocol was employed to measure the intracerebral metabolites (parameters: voxel of interest = 8 × 8 × 8 mm^3^, TR = 2,000 ms, TE = 135 ms, and repetitions = 128). After zero-filling, multiplication with a filter, and eddy-current correction, signal intensities of lactate and N-acetylaspartate were measured from the proton-spectra of metabolites. MRI and MRS quantitative measures were calculated using Mistar (Apollo Imaging Technology, Melbourne, Australia) and Siemens Syngo (Siemens Medical Systems). Based on previous studies (14) and clinical relevance (15) regions of interest (ROIs) were basal ganglia (BG), thalamus (TH), and white matter (WM). The mean value of the specific area bilaterally was calculated for each piglet and used as the measure for statistical analysis. Image data was analysed without knowledge of the randomization category of the piglets. Piglets were euthanized with a lethal dose of pentobarbital following brain imaging. The brain was removed from the skull and fixed by immersion in 4% paraformaldehyde in phosphate buffered saline. Coronal paraffin embedded blocks from the formalin fixed brain tissue were sectioned at 5 µm and stained with hematoxylin and eosin (H&E). Basal ganglia, thalamus, hippocampus, cerebellum, brainstem, and cortex were examined. MRI/MRS and histology evaluations were performed blinded to RIPC treatment group. We correlated NfL values with a) Brain Lactate/N-acetylaspartate ratio (Lac/NAA) measured by magnetic resonance spectroscopy at 72 h in basal ganglia (BG), thalamus (TH), and white matter (WM), and b) Neuropathology score with the extent of damage on hematoxylin and eosin staining in basal ganglia, thalamus, hippocampus, cerebellum, brainstem, and cortex. The extent of damage in each region was graded on a 9 step scale (between 0 and 4 and intervals of 0.5), using a previously published grading system (16).

### Statistics and bioinformatics

Physiologic characteristics were summarized by descriptive statistics, reported as medians, quartiles, and range. Data was tested for normality using D’Agostino-Pearson normality test and inspection of QQ plots. Skewed data were log (ln) transformed for statistical analysis (NfL, Lac/NAA ratios, Neuropathology scores). NfL levels at different timepoints and were compared using repeated measures ANOVA/mixed effects model with Tukey test correction for multiple comparisons. Two group comparisons of parametric data were analysed by *t*-test. Reverse transformed NfL levels were also reported.

Receiver operating characteristic (ROC) curves were created, comparing NfL-2 h with NfL-base and NfL-72 h with NfL-base. Cut-off levels for the ROC curves were obtained where sensitivity and specificity were the highest. Sensitivity and specificity were provided with 95% confidence intervals (CIs).

Multiple Linear regression with NfL-2 h, then NfL-72 h, as the dependent variable was then used to assess the influence of sex and weight on NfL levels.

We used all available serum samples from the main study; therefore, no sample size calculation was performed. A *post hoc* power calculation gave a power of 99,9% to detect an effect size of HI on serum NfL levels of 1 standard deviation with a two-sided statistical significance level of 0.05. Statistical analysis was performed with GraphPad Prism 9 and Stata 14, Post hoc power calculation in G*power 3.1.

## Results

### Physiological measures and HI insult characteristics

Fifty-two piglets were included in the study, of which 48 were subjected to global HI and 4 shams to anaesthesia and normoxic mechanical ventilation only. Piglet characteristics are shown in Table 1. Forty-five minutes of global hypoxia resulted in hypotension, aEEG suppression, and lactic acidosis.

**Table 1:** Physiologic variables.

Time (Group)	Baseline (HI + sham)	Insult (HI)	2 h post HI (HI)	72 h post HI (HI)	72 h sham (sham)
Heart rate (bpm)	144 (18)	187 (35)	176 (24)	144 (22)	157 (8)
MABP (mmHg)	50 (6)	36 (13)	48 (7)		
MABP < 70%* (min)		5.5 (0–15)			
aEEG < 7_u_V (min)		40 (30–43)			
Temperature (°C)	38.6 (0.4)	38.6 (0.4)	38.5 (0.3)	38.3 (0.5)	38.7 (0.1)
pH	7.51 (0.06)	6.98 (0.17)	7.49 (0.07)	7.41 (0.10)	7.51 (0.08)
Lactate (mM)	2.0 (0.9)	17 (3.9)	3.4 (1.8)	2.9 (1.4)	1.2 (0.2)
P_a_CO_2_ (kPa)	5.2 (1)	7.0 (3.5)	4.7 (0.7)	6.1 (1.5)	4.9 (0.6)
Base excess (mM)	7.6 (2)	−17.6 (5.5)	3.8 (3)	1.5 (3.7)	6.3 (2.6)
Glucose (mM)	6.4 (2)	11.7 (4)	7.7 (3.1)	5.8 (1.2)	5.5 (0.1)

Values are means (SD) for normally distributed data or median (IQR) for non-normally distributed data.

MABP, Mean arterial blood pressure.

^a^ MABP < 70%: duration of mean arterial blood pressure below 70% of baseline value.

### Temporal profile of NfL after hypoxia-ischemia

**Figure 1:**
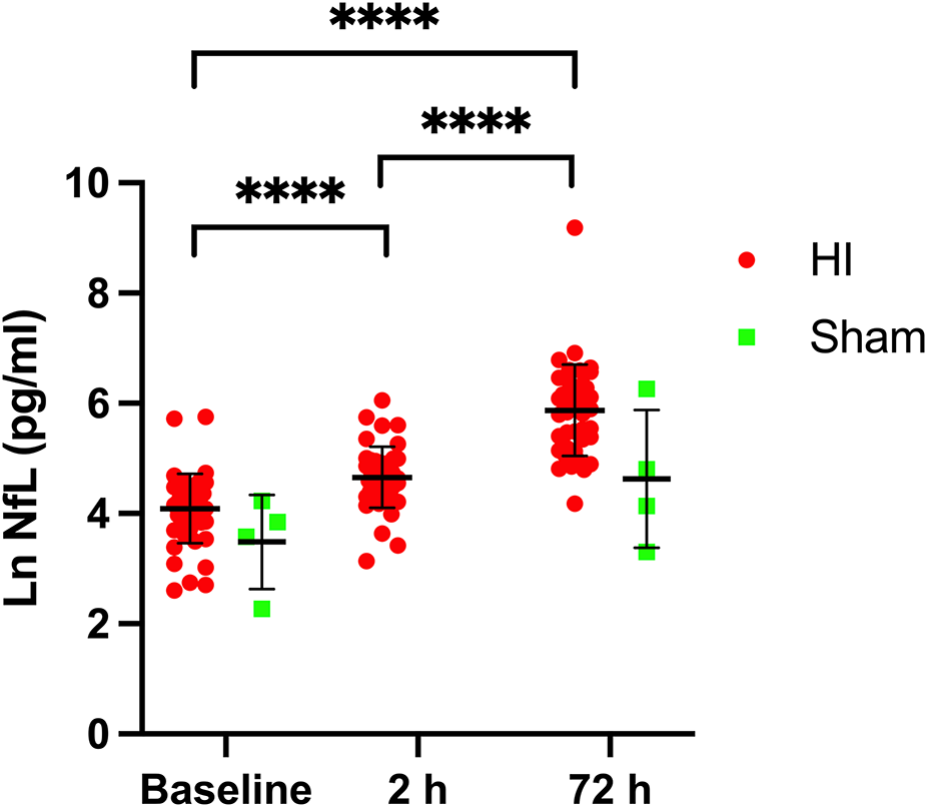
Nfl serum levels at baseline/pre-HI, 2 h after HI and 72 h after HI (red circles) and in sham animals (green squares). ****p < 0.0001, repeated measures ANOVA/mixed effects model with tukey test correction for multiple comparisons. Lines represent means and errorbars are standard deviations.

NfL levels increased significantly by time after HI (Figure 1). The median NfL increase 2 h after HI (NfL-2 h – NfL-base) was 37 pg/ml (95% CI, 25–47, *p* < 0.0001), and at 72 h after HI (NfL-72 h – NfL-base) it was 283 pg/ml (95% CI, 151–423, *p* < 0.0001). Median (IQR) NfL levels were: baseline (*n* = 52) 66 pg/ml (45–87), 2 h (*n* = 45) 105 pg/ml (77–140), and 72 h (*n* = 46) 380 pg/ml (202–552). To test for the influence of time, anaesthesia, and mechanical ventilation NfL was measured in 4 sham piglets not subjected to HI. Median NfL levels were 41 pg/ml (10, 69, 36, and 47 pg/ml) at baseline and 93 pg/ml (27, 63, 122, and 526 pg/ml) after 72 h. In the four sham piglets the median NfL difference from baseline to 72 h was 52 pg/ml (95% CI, −6–479, *p* = 0.11).

### ROC curves for NfL values identifying hypoxia-ischemia exposure

NfL values after HI were compared with baseline pre-HI values.

**Figure 2:**
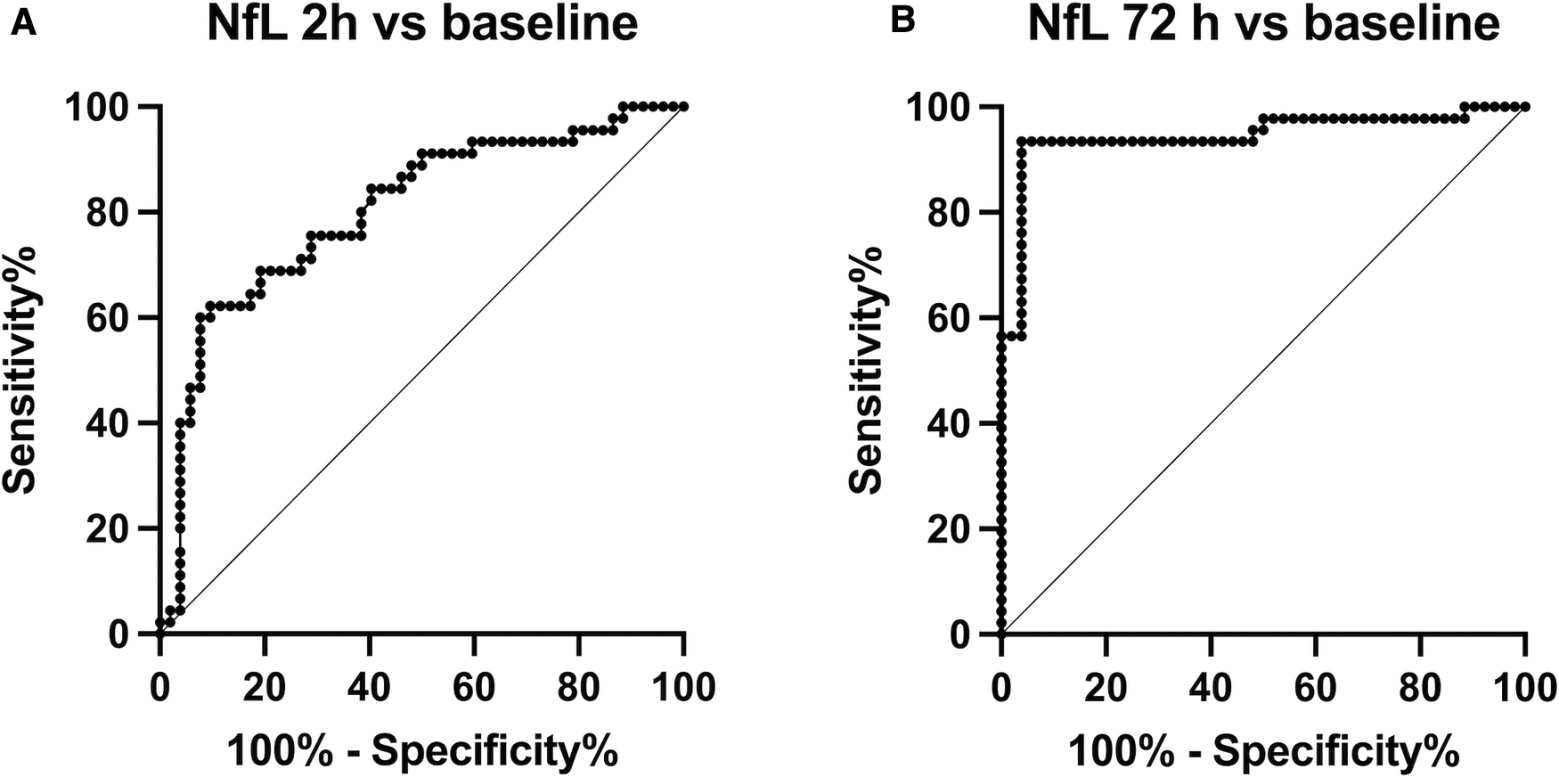
Receiver operating characteristic (ROC) curves, (**A**) comparing NfL-2 h with NfL-base and (**B**) NfL-72 h with NfL-base.

At two hours a cut-off of NfL-2 h > 70 pg/ml would identify piglets exposed to moderate-severe HI in this model of HI-induced brain injury with a sensitivity of 80% (95% CI, 66%–88%) and a specificity of 56% (95% CI, 42%–69%). Likelihood ratio 1.81. Area under the curve 0.76, *p* < 0.0001 (Figure 2A).

At 72 h a cut-off of NfL-72 h > 118 pg/ml would identify piglets exposed to moderate-severe HI in this model of HI-induced brain injury with a sensitivity of 98% (95% CI, 89%–99%) and a specificity of 96% (95% CI, 86%–99%). Likelihood ratio 23.5. Area under the curve 0.97, *p* < 0.0001 (Figure 2B).

### Influence of sex and weight on NfL

Multiple linear regression was used to test if sex and weight significantly predicted NfL-baseline values. The sham group included 2 males and 2 females, piglets subjected to HI were 22 males and 26 females. The fitted regression model was: Ln(NfL-base) ∼ Intercept + sex + wt. The overall regression was statistically significant [*R*^2^ = 0.13, F (2,49) = 3.7, *p* = 0.03]. Sex did not significantly predict Ln(NfL-base) ß = −0.1, *p* = 0.4), while weight significantly predicted Ln(NfL) (ß = −0.0007, *p* = 0.01). Multiple linear regression was then used to test if sex and weight significantly predicted NfL-72 h. The fitted regression model was: Ln(NfL-72 h)∼Intercept + sex + wt. The overall regression was not statistically significant (R^2^ = 0.1, F (2,43) = 2.4, *p* = 0.1. Neither sex (ß = 0.3, *p* = 0.2) nor weight (ß = −0.0008, *p* = 0.06) significantly predicted Ln(NfL-72 h).

### Remote ischemic postconditioning (RIPC) and effect on NfL

**Figure 3:**
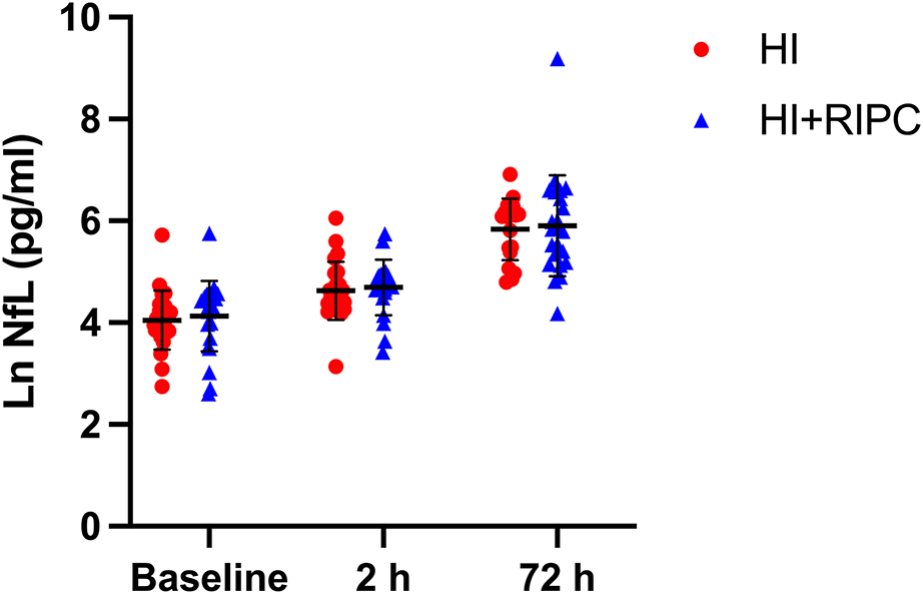
Serum NfL in piglets randomized to supportive treatment only (red circles) or supportive treatment + RIPC (blue triangles) at baseline/pre-HI, 2 h after HI and 72 h after HI. Paired t test revealed no significant difference in NfL levels at any time point for piglets treated with RIPC vs. supportive treatment only (*p* = 0.58 at 72 h). Lines represent means and errorbars are standard deviations.

NfL levels are shown separately for piglets randomized to +/− RIPC in Figure 3. Ln(NfL) group mean differences between HI + RIPC (*n* = 24) and HI groups (*n* = 24) were 0.1 (95% CI, −0.2–0.4) at baseline, 0.1 (95% CI, −0.2–0.4) at 2 h post-HI and 0.1 (95% CI, −0.5–0.6) at 72 h post-HI.

### Correlation between NfL, brain Lac/NAA ratio and neuropathology

**Figure 4:**
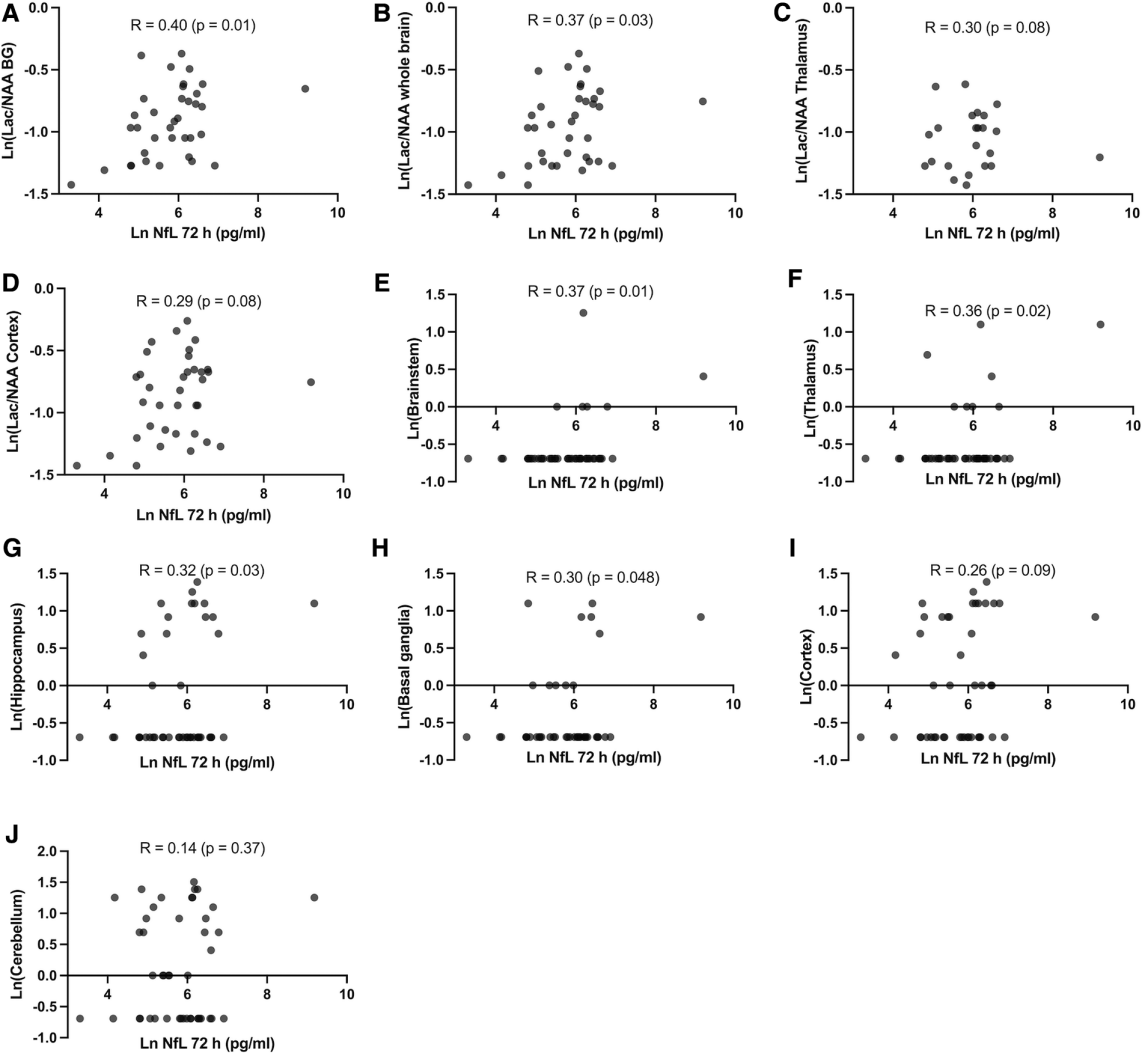
(**A–D**) Pearson's correlation between NfL at 72 h and brain magnetic resonance spectroscopy Lac/NAA ratio in different brain regions at 72 h after HI and (**E–J**) Pearson's correlation between NfL at 72 h and neuropathology score in different brain regions at 72 h after HI.

**Figure 5:**
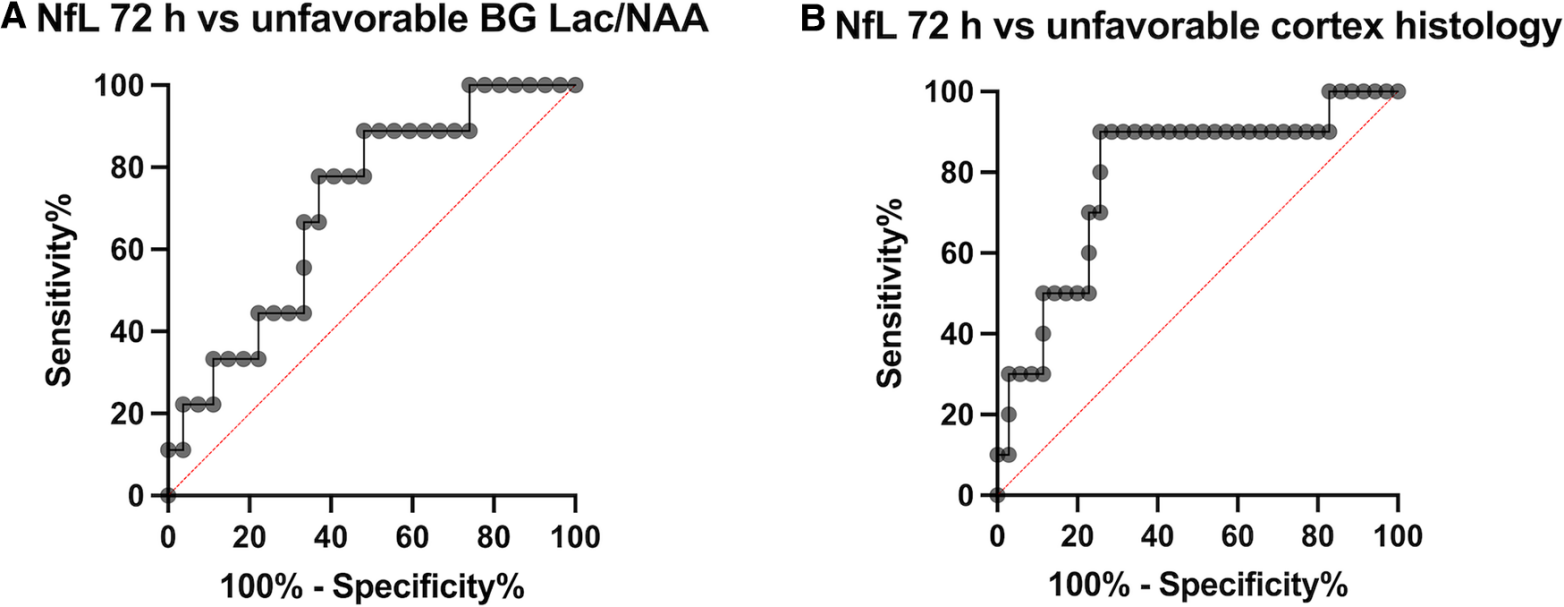
Receiver operating characteristic (ROC) curves, (**A**) comparing NfL-72 h with an unfavourable imaging outcome with basal ganglia Lac/NAA ratio in the worst quartile and (**B**) NfL-72 h with unfavourable outcome with cortex histopathology in the worst quartile.

NfL at 72 h was moderately positively correlated with brain Lac/NAA ratios (Pearson's *r* = 0.29–0.40) and histologic brain injury (Pearson's *r* = 0.14–0.36). (Figure 4). Based on the scatterplots we examined the performance of LnNfL-72 h to predict an unfavourable imaging outcome with basal ganglia Lac/NAA ratio in the worst quartile; Area under the ROC curve was 0.71 (*p* = 0.065). LnNfL-72 h > 5,8 (NfL72 > 330 pg/ml) had a sensitivity of 89% (95% CI, 57%–99%) and a specificity of 52% (95% CI, 34%–69%) for predicting basal ganglia Lac/NAA ratio in the worst quartile (likelihood ratio 1.85) (Figure 5A). We then examined the performance of LnNfL-72 h to predict an unfavourable outcome with cortex histopathology in the worst quartile; Area under the ROC curve was 0.79 (*p* = 0.005). LnNfL-72 h > 6.1 (NfL72 > 445 pg/ml) had a sensitivity of 90% (95% CI, 60%–99%) and a specificity of 74% (95% CI, 58%–86%) for predicting cortical histopathology in the worst quartile (likelihood ratio 3.5) (Figure 5B).

## Discussion

Our main finding was that serum NfL levels increase after newborn HI in a standardized experimental model. The severity of injury in this study was designed to mimic moderate to severe HIE. Insult parameters listed in Table 1 indicate an insult severity comparable to that of similar models with respect to duration of both aEEG suppression (∼40 min) and hypotension (∼10 min), and lactate (∼16 mM) and pH (∼7.0) at end of insult (13, 17).

We identified cut-off NfL levels at 2 h (70 pg/ml) and 72 h (118 pg/ml) after HI for identifying piglets exposed to moderate-severe HI with the associated risk of brain injury. The highest sensitivity and specificity were at 72 h. NfL at 72 h was moderately positively correlated with histologic brain injury and brain Lac/NAA ratios, indicating that the rise in NfL correlates with brain injury after HI.

### Temporal profile of NfL after hypoxia-ischemia

Our data support previous findings that plasma NfL levels increase with time after HI. Thus, the exact timing of NfL sampling is important to interpretation and should be reported in any future trials. Shah et al. found that NfL cut-off levels associated with adverse neurodevelopmental outcomes increased from 28 pg/ml at 18 h after perinatal HI to 436 pg/ml at 98 h after HI (18). Time after birth alone may also have an effect; Depoorter et al. showed that NfL plasma concentrations in healthy preterm and term infants increase after birth from 18.2 pg/ml in umbilical cord blood to 50.9 pg/ml on day 3 (5) which is in part related to delivery mode (6) and might in addition be related to transition from the intra-uterine environment to extra-uterine life. To explore a possible contribution in our model of time after birth without HI, we analysed serum samples from four control piglets not subjected to HIE, but to anaesthesia and mechanical ventilation only. In one sham animal the NfL level increased to 526 pg/ml at 72 h without exposure to hypoxia-ischemia. As none of the sham animals had evidence of brain injury on histology or MRS, this demonstrates that NfL increases may also be seen in the absence of brain injury. It is possible that physiological stress resulting from mechanical ventilation or sedation may have influenced this NfL increase, although this does not explain the variation between sham animals. Median NfL levels increased from baseline to 72 h in sham piglets, but this was only about 20% of the increase seen in HI piglets and was not statistically significant. This indicates that NfL changes observed after HI were primarily the result of the HI-insult, although the small sample size means this should be investigated further in future studies.

### NfL values identifying moderate-severe hypoxia-ischemia exposure

Clinically, there is a continuous spectrum of neurological injury after HI ranging from mild to moderate and severe. Nevertheless, in some cases HIE remains difficult to diagnose. Depending on the setting, only an estimated 50% of neonatal encephalopathy after birth is caused by hypoxia-ischemia, which again may result from a number of events with different timing such as umbilical cord prolapse, obstructed labour, uterine rupture or bleeding (3). Therefore, the ability to identify HI exposure using a biomarker such as NfL is of clinical importance. In our study the PPV at 2 h after HI was 65%, increasing to a PPV of 98% at 72 h after HI. Although the sensitivity of 80% at 2 h was reasonable, the specificity of an early NfL value is only 56%. One interpretation is that early NfL could support the decision to initiate neuroprotective treatment, but only for treatments with relatively few side effects. Existing data on early NfL include umbilical cord blood levels in asphyxiated babies (*n* = 10, of which 3 developed HIE) at 39.2 pg/ml compared to controls (*n* = 18) at 23.3 pg/ml (19). Phosphorylated- Neurofilament heavy chain median plasma levels were 440 pg/ml in infants diagnosed with HIE (*n* = 14, 0–72 h after birth) vs. 15 pg/ml in healthy controls (*n* = 14) (20). We found that NfL is most robust at identifying HI exposure at 72 h after HI. While NfL as a marker of neuronal injury at 72 h after HI does not have bearing on current neuroprotective treatments, there may still be important roles in evaluation of treatment effect, correlations with prognosis and potential implications for future treatments and follow-up.

### Influence of sex, weight, and treatment with RIPC on NfL

A previous study found that NfL levels were higher in low birthweight preterm babies (5). In our data, piglet sex did not influence NfL levels. We found a very small (not biologically important) influence of birthweight on baseline NfL levels, but not NfL at 72 h after HI. In this study, NfL levels were not significantly different at any time point for piglets treated with RIPC. RIPC was induced by occlusion of blood flow to both hind limbs in 4 cycles of 5 min, based on RIPC protocols used in adult clinical studies (21). There are indications however, that neuroprotection in newborn animal models may require a longer duration of RIPC, as neonatal skeletal muscle and its endothelium may be more tolerant of low flow. In a 2021 review of preclinical studies of RIPC after newborn HI by our group, four out of five studies used 4 cycles of 10 min RIPC (22). Thus, RIPC amelioration of serum NfL derived from axonal disruption may require a longer RIPC cycle duration.

### Correlation between NfL, brain Lac/NAA ratio and neuropathology

We found that NfL at 72 h was moderately positively correlated with histologic brain injury and brain Lac/NAA ratios, and that NfL levels are predictive of unfavourable outcomes in basal ganglia Lac/NAA ratios, and cortical histologic brain injury (Figures 4, 5). The correlation between NfL and Lac/NAA is of notice, as Lac/NAA has been shown to be a good predictor of TUNEL positive cell death in a series of piglet studies of newborn HI (23). Lac/NAA also has a high correlation with neurodevelopmental outcomes in clinical studies (24, 25). In comparison, Apgar score at 10 min does not predict the risk of death or moderate or severe disability well, with an AUC of 0.56 for Apgar 0 at 10 min (26). A systematic review found that amplitude integrated EEG (aEEG) background pattern had a pooled sensitivity of 0.90 and specificity of 0.46 for predicting an unfavourable outcome on clinical follow-up (27). A maximal clinical Thompson-score >11 in the first 10 days of life has been reported to have a sensitivity of 0.93 and specificity of 0.61 (AUC 0.84) for predicting survival without cognitive impairment (28). aEEG and Thompson score are both continuously assessed beyond 72 h, suggesting that there may be a role for NfL as a moderately early predictor of brain damage.

Our results on NfL are in keeping with data published on 26 term babies with HIE treated with hypothermia. NfL > 29 pg/ml less than six hours after HI was predictive of an unfavourable outcome on MRI, with a sensitivity of 77% and specificity of 69% (29). During therapeutic hypothermia on day 3 a cut-off of 166 had a sensitivity of 82% and specificity of 69%. After rewarming on day 5 an NfL level of >417 pg/ml had a sensitivity of 92% and a specificity of 92% for unfavourable outcome on MRI. On clinical follow-up at 18 months NfL cut-off levels of >436 pg/ml on day 5 after rewarming was associated with adverse outcome with a diagnostic sensitivity of 75%, specificity 77%, PPV 75%, and NPV 77%. NfL levels at earlier time points were not predictive of outcome (18). Thus, there are indications that NfL is associated to long-term prognosis rather than just acute neuronal injury. This is supported by a study in preterm infants with peri/intraventricular haemorrhage, which found that serum NfL levels correlated with motor outcomes at one and two years of age (30).

### Strengths and limitations

A major advantage of the study is that each piglet´s baseline value served as its own control. This approach allowed for paired intra-individual comparison before and after HI, excluding competing aetiologies, and limiting genetic and environmental confounding. Further, as the timing and severity of HI is controlled, this increases our knowledge of serum NfL dynamics after HI and should contribute to the interpretation of future clinical studies. In accordance with the 3R's principle (Replacement, Reduction, Refinement) for animal experimentation (31), the animals in the study were also used to investigate other outcomes in a previously published study (10). For this reason, an *a priori* power calculation was not performed. Important limitations include the translatability of the model, specifically with regards to serum NfL in the piglet. However, the absolute serum NfL values measured in our study are in the same range as those reported in infants with brain injury (30). The small sample size in the sham group means that findings in this group must be interpreted with caution. Further, the mean age of piglets at experiment start was 22 h, which should be considered when interpreting NfL changes over time. Four sham animals were included to investigate the effects influence of time, anaesthesia, and mechanical ventilation. The 72-hour NfL of 526 pg/ml in one sham animal highlights the risk that anaesthesia or other procedures may in themselves increase NfL. In HI animals it is also possible that anaesthesia impacts evolving brain injury, as anaesthetic gasses are thought to be neuroprotective. Anaesthesia could thus result in lower NfL levels than might otherwise be seen. However, anaesthesia is an integral part of the model and previous studies have demonstrated relevant brain injury with anaesthesia (10, 12). We did not investigate NfL metabolism, and to our knowledge the metabolism of NfL in cerebrospinal fluid (CSF) and blood is largely unknown, with no description of ways of elimination or protein degradation (5). CSF NfL levels have been reported to be 113-fold higher than serum levels in preterm infants with peri/intraventricular haemorrhage (30). With regards to the identified cut-off levels for identifying HI exposure and associated sensitivities and specificities this data driven approach may lead to overly optimistic measures of diagnostic accuracy (32). Further, the ROC curves are based on baseline vs. post-HI values, rather than post-HI vs. sham values from identical time points. This comparison ignores that increases over time may not only reflect HI, but likely also time-related changes, which in the experimental model might be attributable to surgery, anaesthesia, housing environmental stress, or natural development after birth. Taken together, these limitations mean that the sensitivity and specificities for NfL as a marker of HI are likely to be significantly overestimated in our analysis. More robust values require testing in future separate studies with larger sample sizes including comparison with a larger sham group.

### Conclusion

We have shown that serum NfL values increase after HI in clinically relevant model of perinatal hypoxia-ischemia. The diagnostic sensitivity and specificity for the moderate-severe HI induced in this model was highest at 72 h after HI, and at 72 h NfL values were predictive of a poor outcome on cortical histopathology and magnetic resonance spectroscopy basal ganglia Lac/NAA ratio. Further studies are needed to investigate NfL dynamics over time and how NfL values correspond to short and long-term neurologic impairment in infants with varying degrees of hypoxic ischemic injury. NfL could potentially provide a biological link to identify brain injury after perinatal hypoxia-ischemia.

## Data Availability

The raw data supporting the conclusions of this article will be made available by the authors, without undue reservation.
